# Failure to Deliver and Translate—New Insights into RNA Dysregulation in ALS

**DOI:** 10.3389/fncel.2017.00243

**Published:** 2017-08-17

**Authors:** Alyssa N. Coyne, Benjamin L. Zaepfel, Daniela C. Zarnescu

**Affiliations:** ^1^Department of Molecular and Cellular Biology, University of Arizona Tucson, AZ, United States; ^2^Department of Neuroscience, University of Arizona Tucson, AZ, United States; ^3^Department of Neurology, University of Arizona Tucson, AZ, United States

**Keywords:** RNA transport, stress granules, translation, TDP-43, FUS, C9orf72

## Abstract

Amyotrophic Lateral Sclerosis (ALS) is a progressive and fatal neurodegenerative disease affecting both upper and lower motor neurons. The molecular mechanisms underlying disease pathogenesis remain largely unknown. Multiple genetic loci including genes involved in proteostasis and ribostasis have been linked to ALS providing key insights into the molecular mechanisms underlying disease. In particular, the identification of the RNA binding proteins TDP-43 and fused in sarcoma (FUS) as causative factors of ALS resulted in a paradigm shift centered on the study of RNA dysregulation as a major mechanism of disease. With wild-type TDP-43 pathology being found in ~97% of ALS cases and the identification of disease causing mutations within its sequence, TDP-43 has emerged as a prominent player in ALS. More recently, studies of the newly discovered *C9orf72* repeat expansion are lending further support to the notion of defects in RNA metabolism as a key factor underlying ALS. RNA binding proteins are involved in all aspects of RNA metabolism ranging from splicing, transcription, transport, storage into RNA/protein granules, and translation. How these processes are affected by disease-associated mutations is just beginning to be understood. Considerable work has gone into the identification of splicing and transcription defects resulting from mutations in RNA binding proteins associated with disease. More recently, defects in RNA transport and translation have been shown to be involved in the pathomechanism of ALS. A central hypothesis in the field is that disease causing mutations lead to the persistence of RNA/protein complexes known as stress granules. Under times of prolonged cellular stress these granules sequester specific mRNAs preventing them from translation, and are thought to evolve into pathological aggregates. Here we will review recent efforts directed at understanding how altered RNA metabolism contributes to ALS pathogenesis.

## Introduction

Amyotrophic Lateral Sclerosis (ALS) is a progressive and fatal neurodegenerative disease. Affecting both upper and lower motor neurons, ALS culminates in the inability to coordinate muscle movements with death due to respiratory failure typically within 2–5 years after diagnosis (Andersen and Al-Chalabi, 2011; Robberecht and Philips, 2013). Although clinically indistinguishable, 10% of ALS cases are classified as familial (FALS) and the remaining 90% are sporadic (SALS; Andersen and Al-Chalabi, 2011; Ling et al., 2013; Robberecht and Philips, 2013).

Although, ALS is primarily a motor neuron disease, it belongs to a spectrum of neurodegenerative diseases encompassing ALS and the related Fronto-Temporal Dementia (FTD). About 50% of ALS patients display deficits reminiscent of FTD and 15% of FTD patients have ALS symptoms (Ringholz et al., 2005; Wheaton et al., 2007; Ferrari et al., 2011). Consistent with these observations are findings that several genetic loci implicated in ALS have also been linked to FTD (Ling et al., 2013; Robberecht and Philips, 2013).

In 1993, mutations in the gene encoding Superoxide Dismutase (SOD1) were the first to be linked to ALS pathogenesis (Rosen et al., 1993). Since that time, multiple other genetic loci have been linked to ALS including those involved in proteostasis, cytoskeletal organization and RNA metabolism (Robberecht and Philips, 2013; Peters et al., 2015). Furthermore, both genetic and environmental risk factors have been linked to ALS (Zufiría et al., 2016). However, the contribution of the environment to ALS pathology remains correlative and poorly understood. Based on the known functions of the ALS loci, multiple disease pathomechanisms have been proposed (Lagier-Tourenne and Cleveland, 2009; Robberecht and Philips, 2013). Notably, initial hypotheses following the discovery of SOD1 were centered around altered proteostasis (Morimoto et al., 2007; Sasaki, 2011). However, the identification of TAR DNA-binding protein 43 (TDP-43), fused in sarcoma (FUS), and C9orf72 has led to a paradigm shift highlighting the dysregulation of RNA metabolism as a critical, causative factor in ALS/FTD (Ling et al., 2013).

Dysregulation of RNA metabolism can occur at multiple levels of RNA processing including transcription, splicing, mRNA transport, stability and translation (Lagier-Tourenne and Cleveland, 2009; Ling et al., 2013; Robberecht and Philips, 2013). In addition, TDP-43, FUS, and repeat-associated non-AUG (RAN) translated *C9orf72* dipeptide repeat proteins (DPRs) associate with stress granules under times of cellular duress (Bentmann et al., 2013; Dafinca et al., 2016; Lee et al., 2016; Maharjan et al., 2017). Stress granules sequester proteins and mRNAs, and inhibit translation of specific transcripts to allow for the cell to recover from stress (Anderson and Kedersha, 2009; Buchan, 2014; Protter and Parker, 2016). It has been proposed that aberrant formation and persistence of stress granules can seed the formation of pathological aggregates in disease (Dewey et al., 2012; Aulas and Vande Velde, 2015). As a result, proteins and mRNAs become trapped and unable to perform their normal functions, which in turn leads to defects in RNA processing and expression (e.g., altered ribostasis Ramaswami et al., 2013).

**Figure 1:**
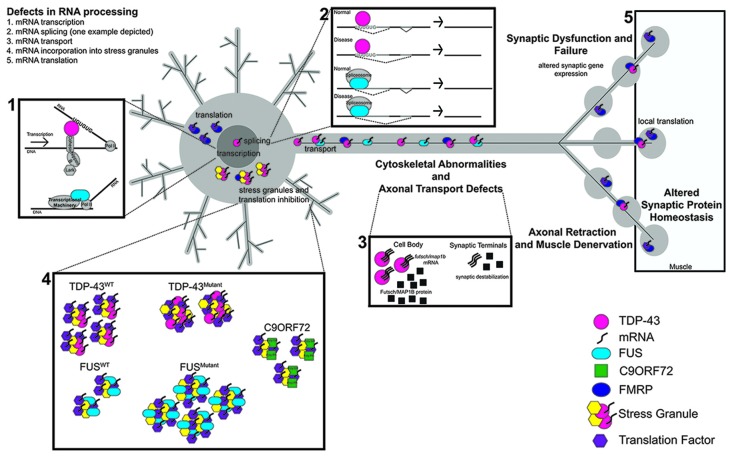
The RNA Binding proteins TAR DNA-binding protein 43 (TDP-43) and fused in sarcoma (FUS) function in several aspects of RNA metabolism. **(1)** TDP-43 regulates the transcription of UG repeat containing genes by stabilizing the binding of cohesin and nipped B to DNA. FUS binds to RNA Pol II to regulate the transcription of target genes. **(2)** Both TDP-43 and FUS regulate the splicing of target genes; TDP-43 via its interaction with UG repeats and FUS via its interaction with components of the spliceosome. **(3)** In disease, defects in RNA processing at any stage lead to transport defects (nucleo/cytoplasmic, axonal). **(4,5)** TDP-43 and FUS also alter stress granule dynamics, which can in turn impact mRNA translation. Dipeptide repeat proteins (DPRs) from *C9orf72* repeat expansions have also been shown to associate with stress granules. Synaptic dysfunction and failure likely result from defects in RNA processing. Proteins and RNA as indicated. *C9orf72* collectively represents G_4_C_2_ repeats and DPRs.

As ALS progresses, multiple cellular processes are disrupted, ultimately leading to synaptic failure and muscle denervation (Robberecht and Philips, 2013). However, the mechanisms underlying ALS pathogenesis remain poorly understood. Elucidating primary defects in RNA metabolism that lead to ALS pathogenesis is critical for our understanding of disease progression. Key questions in the field include: (1) what are the protein partners and RNA targets that act as effectors of toxic phenotypes in disease? (2) Are there shared targets among disease associated mutations? (3) How might otherwise distinct mutations converge to produce the same phenotypic outcome across FALS and SALS? Although several other RNA binding (e.g., senataxin, hnRNPA1 Chen et al., 2004; Kim et al., 2013) or stress granule associated proteins (e.g., TIA-1, profilin Liu-Yesucevitz et al., 2010; Wu et al., 2012; Figley et al., 2014) have been linked to ALS/FTD, here we will address some of these questions by reviewing recent literature on RNA metabolism in ALS and discuss how dysregulation of RNA processing steps may contribute to disease from the perspective of three critically important genes, namely *TARDP*, *FUS* and *C9orf72* (Figure 1).

## RNA Binding Proteins in ALS

### TAR DNA Binding Protein (TDP-43)

TDP-43, encoded by the *TARDBP* gene, is a DNA and RNA binding protein containing nuclear localization and export signals (NLS, NES), two RNA recognition motifs (RRM1-2), and a glycine rich C-terminal prion like domain (Ou et al., 1995; Cushman et al., 2010; King et al., 2012). In animal models based on overexpression, RNA binding and the RRM1 domain are required for toxicity (Voigt et al., 2010; Ihara et al., 2013) lending support to the notion that dysregulation of RNA metabolism contributes to disease. While the RRM domains mediate interactions with RNA and DNA, the C terminal domain mediates protein–protein interactions (Budini et al., 2014). Following the identification of wild-type TDP-43 as a component of pathological aggregates (Neumann et al., 2006), point mutations were discovered to be causative of ALS (Kabashi et al., 2008; Sreedharan et al., 2008). Interestingly, the majority of ALS-associated mutations in *TARDBP* lie within the unstructured C terminal domain and have been shown to increase TDP-43’s intrinsic propensity to aggregate *in vitro* and in yeast (Johnson et al., 2009; Jiang et al., 2016; Lim et al., 2016). As wild-type TDP-43 pathology is linked to ~97% of ALS cases regardless of etiology (Ling et al., 2013), understanding both the normal function and involvement in disease for both wild-type and mutant TDP-43 is crucial to understanding ALS pathogenesis.

#### Transcription, Splicing and RNA Stability

*In vitro* binding and RNA seq approaches have revealed that TDP-43 binds predominantly UG rich sequences and regulates the expression and splicing of multiple targets (Buratti and Baralle, 2001; Polymenidou et al., 2011; Sephton et al., 2011; Tollervey et al., 2011; Narayanan et al., 2013). TDP-43 has been shown to repress the transcription of both the HIV-1 TAR DNA element *in vitro* (Ou et al., 1995) and the testis-specific mouse *acrv1* gene *in vivo* (Lalmansingh et al., 2011). In addition, in mammalian neurons, TDP-43 localizes to sites of transcription and splicing within the nucleus and is absent from areas of silent heterochromatin (Casafont et al., 2009). Recently, it was shown that mechanistically, TBPH, the *Drosophila* homolog of TDP-43, binds to nascent RNA transcripts that contain UG repeats. This stabilizes the binding of chromatin regulators such as cohesin and Nipped B to DNA thus, facilitating transcription of specific target genes (Swain et al., 2016).

Interestingly, TDP-43 differentially regulates the expression of TIA-1 and G3BP (McDonald et al., 2011), both of which are core components of stress granules (Kedersha and Anderson, 2007). Reducing TDP-43 by siRNA causes an increase in TIA-1 and a decrease in G3BP mRNA, and protein expression in HeLa cells. These effects are accompanied by a reduction in stress granule number and size (McDonald et al., 2011). Given that acute, non-persistent stress granules are thought to be protective (reviewed in Wolozin, 2012), this dysregulation could impact a cell’s ability to recover from even normal levels of cellular stress.

TDP-43 was also shown to function as a splicing regulator of the CFTR gene where it controls exon skipping by binding to UG repeats within the pre-mRNA (Buratti and Baralle, 2001; Buratti et al., 2004). Interestingly, TDP-43’s many protein partners include those involved in pre-mRNA splicing (Freibaum et al., 2010; Sephton et al., 2011). In addition, loss of TDP-43 expression influences the splicing of multiple RNA targets (Polymenidou et al., 2011; Tollervey et al., 2011).

Mice expressing disease-associated mutant TDP-43^Q331K^, which remains primarily restricted to the nucleus, display evident alterations in splicing of predominantly direct targets of TDP-43. While for some RNA targets the Q331K mutation appears to mimic a loss of normal TDP-43 splicing function, for others a gain of splicing function was observed (Arnold et al., 2013). In contrast, studies in mammalian cell lines, human ALS fibroblasts or primary neuronal cultures, of disease associated TDP-43 mutants show that mutant variants appear to largely retain their splicing activity (Budini et al., 2012; Colombrita et al., 2015). Possible explanations for this apparent discrepancy are that different mutations in TDP-43 may have different effects and that TDP-43 may have a different repertoire of mRNA targets in distinct cell types used for these studies. Supporting the latter possibility, specific loss of TDP-43 resulted in a different repertoire of cryptic exons in excitatory neurons compared to skeletal monocytes (Jeong et al., 2017).

A hallmark of ALS is the mislocalization of TDP-43 from the nucleus to the cytoplasm, which suggests that a nuclear loss of function and cytoplasmic gain of function may contribute to ALS pathogenesis. Indeed, cytoplasmic expression of TDP-43 is toxic, causing alterations in the expression of genes that encode factors regulating chromatin assembly and the function of lysosomes among others (Barmada et al., 2010; Amlie-Wolf et al., 2015). Expression of TDP-43 C-terminal fragments in cells results in a decrease in CFTR exon 9 skipping similar to what was observed for TDP-43 loss of function (Igaz et al., 2009). Significant alterations in RNA splicing and the spliceosome have been found in post-mortem lower motor neurons and in mammalian cells where TDP-43 expression was reduced (Highley et al., 2014). In patient fibroblasts expressing mutant TDP-43, similar changes in gene expression and splicing occurred that were distinct from alterations found in sporadic or SOD1 fibroblasts (Highley et al., 2014). Furthermore in a pig model of ALS, mutant TDP-43 interacts with the RNA splicing factor PSF resulting in a mislocalization of PSF and its partner NeuN to the cytoplasm. Consequently, this causes alterations in PSF mediated splicing events that can also be found in ALS patient brain tissue (Wang et al., 2015). Together, these studies suggest that TDP-43 can cause defects in splicing that are either a direct consequence of loss of TDP-43 regulation, or TDP-43 dependent dysregulation of essential components of the splicing machinery.

Many of the genes that are potentially regulated by TDP-43 encode proteins involved in RNA processing or synaptic function (Polymenidou et al., 2011; Sephton et al., 2011; Narayanan et al., 2013). Moreover, TDP-43 has been shown to bind to, and regulate the expression of other genes involved in ALS, FTD, and related neurodegenerative diseases including FUS, Tau, ataxin 2, and progranulin (Polymenidou et al., 2011; Sephton et al., 2011; Tollervey et al., 2011). Interestingly, genes with large intronic regions and those encoding proteins involved in synaptic activity are most affected by loss of TDP-43 in mouse brain (Polymenidou et al., 2011). In flies, loss of TBPH alters the splicing of the calcium channel *cacophony* (Chang et al., 2014) and the expression of syntaxin 1A mRNA and protein (Romano et al., 2014). Moreover, pan-neuronal overexpression of wild-type TDP-43 in flies reduces *marf* mRNA and protein expression, which contributes to the failure of TDP-43 expressing flies to maintain ability to respond to continuous synaptic stimulation (Khalil et al., 2017). Together these data suggest that alterations in genes encoding synaptic proteins may at least in part contribute to the synaptic dysfunction observed in ALS. Furthermore, the transcription or splicing dysregulation of genes involved in RNA processing or disease could exacerbate TDP-43’s effects on RNA metabolism.

TDP-43 autoregulates its expression by binding to the 3′ UTR of its own transcript, which in turn causes RNA instability and degradation (Ayala et al., 2011; Polymenidou et al., 2011). Interestingly, overexpression of wild-type TDP-43 decreases expression of tau, a key protein implicated in both FTD and Alzheimer’s Disease, by decreasing *tau* mRNA stability (Gu et al., 2017). Moreover, loss of TDP-43 results in decreased *beta-adducin 2* mRNA stability (Costessi et al., 2014) suggesting that in addition to direct transcriptional or splicing regulation, TDP-43 may also affect gene expression by controlling the stability of RNA transcripts that at least in these cases may have a direct effect on synaptic function.

#### mRNA Transport

In addition to binding to, and altering gene expression for loci encoding synaptic proteins (Polymenidou et al., 2011; Sephton et al., 2011; Narayanan et al., 2013), TDP-43 can be visualized within mouse synaptic terminals (Narayanan et al., 2013), in fly motor neuron axons (Alami et al., 2014), and in dendrites of cultured hippocampal neurons (Wang et al., 2008), suggesting that TDP-43 may bind to synaptic mRNA transcripts and transport them to distal locations. Indeed, TDP-43 is present in axonal RNP granules that are trafficked in a microtubule dependent manner. In these granules, TDP-43 transports mRNA targets such as neurofilament light (NEFL) mRNA to distal axonal regions (Alami et al., 2014). Elegant live imaging studies showed that disease associated mutations in TDP-43 disrupt axonal trafficking in primary, human cortical and motor neurons (Alami et al., 2014). Furthermore, TDP-43 forms a complex with and alters the localization of *futsch/MAP1B* mRNA in *Drosophila* (Coyne et al., 2014) resulting in synaptic microtubule instability, which could potentially compound effects on axonal mRNA transport. Recently, TDP-43 has been shown to bind G quadruplex forming mRNAs and transport them into distal neurites (Ishiguro et al., 2016). Once again, disease associated mutations in TDP-43 disrupt this process as they no longer bind and transport G quadruplex containing mRNAs. These data suggest that TDP-43 regulates the transport of specific mRNA targets in RNP granules along axonal microtubules for local translation at synaptic sites.

#### Stress Granules and Translation

TDP-43 not only colocalizes with its mRNA targets in neuritic RNP granules (Alami et al., 2014; Ishiguro et al., 2016), but under times of cellular stress and in disease, associates with stress granules (Colombrita et al., 2009; Liu-Yesucevitz et al., 2010; Dewey et al., 2011; Parker et al., 2012). Stress granules are transient, dynamic, membraneless cytoplasmic structures that form in response to cellular stress and result in translation inhibition of most mRNAs except for those involved in cellular recovery (Anderson and Kedersha, 2009). Importantly, TDP-43 associates with stress granules via direct protein—protein and RNA dependent interactions (Liu-Yesucevitz et al., 2010) with the RRM1 domain and C-terminal residues 216–315 both being necessary for TDP-43 incorporation into stress granules (Colombrita et al., 2009).

While TDP-43 is not required for stress granule formation, it alters their dynamics (Dewey et al., 2011; McDonald et al., 2011; Liu-Yesucevitz et al., 2014). Disease associated mutations in TDP-43 increase stress granule size and subsequently reduce the mobility of arsenite induced stress granules in mammalian cells and rat hippocampal neurons (Dewey et al., 2011; Liu-Yesucevitz et al., 2014). Furthermore, disease associated mutations in TDP-43 alter the liquid-like biophysical properties of axonal RNP granules (Gopal et al., 2017). In contrast, when cells are subjected to osmotic stress, wild-type TDP-43 expression does not affect stress granule size, however their numbers increase (Dewey et al., 2011; Liu-Yesucevitz et al., 2014). Although the mechanism by which mutant TDP-43 alters stress granule dynamics remains largely unknown, it is plausible that cytoplasmic mislocalization (Barmada et al., 2010), the interaction between disease associated mutant TDP-43 and stress granule components (Bentmann et al., 2013), and its propensity to aggregate (Johnson et al., 2009) contribute to its pathological accumulation.

It is hypothesized that prolonged exposure to cellular stress in disease leads to the accumulation of cytoplasmic stress granules which may evolve into pathological aggregates (Ramaswami et al., 2013). Supporting this hypothesis are findings that in patient tissues, TDP-43 co-localizes with multiple stress granule markers including TIA-1 and eIF3 (Liu-Yesucevitz et al., 2010). Persistent stress granules and aggregates are thought to sequester RNA binding proteins such as translation initiation factors and TDP-43 as well as their mRNA targets preventing their translation and potentially contributing to disease progression (Ramaswami et al., 2013). Notably, pharmacological inhibition of eIF2α reduces toxicity in *Drosophila* and rat primary neurons (Kim et al., 2014). Given that phosphorylation of eIF2α causes translation initiation complexes to accumulate into stress granules (Thomas et al., 2011), these studies suggest that TDP-43 may induce a cascade of events affecting stress granule formation and kinetics, which can ultimately affect translation.

Consistent with a role in translation, TDP-43 forms a complex with multiple components of the translational machinery in mammalian cells (Freibaum et al., 2010). Furthermore, TDP-43 can associate with the translational machinery via the ribosomal protein RACK1 suggesting that TDP-43 may have a more direct role in translation regulation (Russo et al., 2017). Indeed, TDP-43 regulates the translation of *futsch/MAP1B* mRNA (Coyne et al., 2014). Mechanistically, TBPH binds to the 5’UTR of *futsch* to post-transcriptionally regulate its expression (Romano et al., 2016). Futsch/MAP1B is a microtubule associated protein (Hummel et al., 2000; Roos et al., 2000) and ultimately, alterations in *futsch/MAP1B* mRNA processing lead to microtubule instability at the *Drosophila* neuromuscular synapse (Coyne et al., 2014). Furthermore, TDP-43 and its protein partner FMRP share several mRNA targets including *rac1*, *map1b*, *gluR1* and *sirt1* mRNAs (Yu et al., 2012; Coyne et al., 2015; Majumder et al., 2016). Interestingly, in flies, overexpression of FMRP mitigates TDP-43 induced toxicity by remodeling TDP-43 containing RNP granules and restoring the translation of specific mRNA targets (Coyne et al., 2015). Although TDP-43 may directly bind to and regulate the translation of some of its mRNA targets (Romano et al., 2016), it is likely that the translation of other mRNAs is altered via sequestration into stress granules during disease progression. The mechanism by which TDP-43 may interact with proteins involved in translation initiation to directly regulate or sequester mRNA transcripts remains largely unknown.

While most studies have focused on gene expression changes induced by overexpression or reduction of wild-type TDP-43, the consequences induced by disease associated mutant TDP-43 remain less understood. It has been shown that ALS associated mutations in TDP-43 can disrupt axonal mRNA transport (Alami et al., 2014), RNP transport dynamics (Gopal et al., 2017), alter the splicing of RNA targets (Arnold et al., 2013), and influence stress granule dynamics (Dewey et al., 2011; McDonald et al., 2011; Liu-Yesucevitz et al., 2014). It will be interesting to see in future studies whether wild-type and mutant TDP-43 regulate their mRNA targets via similar or distinct mechanisms.

#### TDP-43 Mediated Effects on RNA Metabolism in Glia

In addition to neuronal inclusions, TDP-43 inclusions have been reported in glia in ALS tissue samples (Zhang et al., 2008). As a result, there has been much speculation and debate about the cell autonomous and non-cell autonomous roles of glial TDP-43 in disease. While some groups report that astroglial expression of mutant TDP-43 has detrimental effects on neuronal survival (Tong et al., 2013; Yan et al., 2014), others do not see any neuronal toxicity resulting from glial expression of TDP-43 (Haidet-Phillips et al., 2013; Serio et al., 2013). It has been suggested that preferential neuronal toxicity observed in some studies may be due to higher TDP-43 expression in neurons compared to glia (Yan et al., 2014).

Studies in *Drosophila* have shown that pan-glial expression of TDP-43 may contribute to ALS phenotypes (Diaper et al., 2013b; Estes et al., 2013; Romano et al., 2015). When expressed in glia, TDP-43 assembles in cytoplasmic puncta that appear to surround the NMJs, causes locomotor dysfunction and a non-cell autonomous increase in post-synaptic glutamate receptors (Estes et al., 2013). TBPH loss of function in glia reduces gliopod-like projections at the NMJ suggesting a normal glial-autonomous role for TDP-43 in axonal wrapping (Romano et al., 2015). Mechanistically, TBPH loss of function causes decreased expression of EAAT1 and EAAT2 glutamate transporter mRNA leading to defects in synaptic function. Glial specific overexpression in a TBPH mutant background rescued locomotor dysfunction and mitigated GluRIIA levels suggesting that altered EAAT1 expression is at least partially responsible for NMJ phenotypes induced by TBPH loss. Additionally, while glial overexpression of TBPH reduces EAAT1/2 expression, neuronal overexpression increases EAAT1/2 expression consistent with cell type specific effects of TDP-43 on gene expression (Diaper et al., 2013a). In rats expressing TDP-43^M337V^ in astrocytes, there is a reduction in EAAT1/2 glutamate transporters in the spinal cord (Tong et al., 2013) while a loss of EAAT2 protein has been reported in ALS patient motor cortex and spinal cord resulting from the presence of abnormally spliced EAAT2 mRNA variants (Bristol and Rothstein, 1996; Lin et al., 1998). To determine TDP-43 mediated changes in glial gene expression, microarray analysis of astrocytes expressing TDP-43^M337V^ revealed a downregulation of neurotrophic genes and conversely, an upregulation of neurotoxic genes (Tong et al., 2013). This provides a possible explanation for non-cell autonomous, neuronal cell death caused by glial TDP-43 toxicity. Further experiments are needed to comprehensively assess glial specific alterations in RNA metabolism induced by TDP-43 and to fully understand the contribution of glia to disease.

### Fused in Sarcoma (FUS)

FUS is an RNA- and DNA-binding protein harboring an RNA binding region, a Cys_2_/Cys_2_ finger domain, and SYQG, RGG, and G rich regions (Morohoshi et al., 1998). Similar to TDP-43, mutations in the RNA binding protein FUS are causative of ALS (Kwiatkowski et al., 2009), accounting for about 5% of fALS cases (Ling et al., 2013). Several FUS mutations lie within the predicted NLS consistent with findings that in disease FUS is mislocalized from the nucleus to the cytoplasm (Kwiatkowski et al., 2009; Vance et al., 2009; Lattante et al., 2013). The most aggressive cases of fALS are caused by the R522G and P525L mutations, which cause a significant redistribution of FUS to the cytoplasm and lead to early disease onset (Dormann et al., 2010). Toxicity resulting from mutations in FUS is thought to be the result of alterations in multiple RNA processing steps including transcription, splicing, incorporation into stress granules, and neuronal RNA localization.

#### Transcription, Splicing and RNA Stability

Knockdown of FUS in mouse spinal cords followed by RNA-seq identified over 600 transcriptional targets, with 61 downregulated and four upregulated more than two-fold (Lagier-Tourenne et al., 2012). A subsequent study using RNA-seq analysis of mouse neurons derived from embryonic stem cells showed that knockdown of FUS leads to differential expression of 146 genes (Nakaya et al., 2013). Common dysregulated genes identified in these studies include synaptic proteins *Neurexin 3* and *Neuroligin-1*, as well as *Kcnip4*, a potassium voltage-gated ion channel-interacting protein (Lagier-Tourenne et al., 2012; Nakaya et al., 2013). Additional studies showed that overexpression or siRNA knockdown of FUS in HeLa cells leads to significantly altered expression of several genes, including the spliceosome mediator *INTS3, methyl CpG binding protein 2* (*MECP2*) and *LISTERIN* (Tan et al., 2012). Interestingly, altered *LISTERIN* expression has previously been linked to the degeneration of white matter in the ventrolateral side of the lumbar spinal cord in mice (Chu et al., 2009). Neurons within this region are involved in locomotion and muscle coordination highlighting the relevance of *LISTERIN* as a target of FUS, as well as its potential role in the pathology of ALS.

FUS regulates the transcription of its target genes via binding to single-stranded DNA (ssDNA) harboring TLS/FUS response elements located in the promoter region (Tan et al., 2012). Loss of FUS in human cells broadly affects transcription, as it causes accumulation of Ser2-phosphorylated RNA polymerase II (RNAP II) at the transcription start site of several genes, leading to stalling and premature termination of transcription. Mechanistically, FUS-RNA binding promotes the assembly of higher order fibrous assemblies that bind to the CTD of RNAP II (Schwartz et al., 2012, 2013). Additionally, FUS indirectly alters transcript levels by modulating the activity of other transcription factors and modifying the stability of target mRNAs (Coady and Manley, 2015; Yamaguchi and Takanashi, 2016). Direct protein interactions between FUS and scaffold attachment factor B1 (SAFB1) in mammalian cells repress androgen-receptor (AR) mediated transcription (Yamaguchi and Takanashi, 2016). While the role of AR-mediated transcription in ALS has yet to be resolved, dysregulation of its activity provides a plausible mechanism by which interactions between FUS and its protein partners may lead to specific transcriptional changes within motor neurons.

Depletion of FUS from iPSC-derived neural progenitor cells led to increased half-life of 330 genes, and decreased half-life of 44 genes, indicating a role for FUS in regulating the stability of target mRNAs (Kapeli et al., 2016). In mammalian cells, mutant FUS increases the stability of *MECP2* mRNA (Coady and Manley, 2015). Interestingly, MECP2 protein levels are reduced, which correlates with mutant FUS mediated accumulation of *MECP2* mRNA in insoluble aggregates (Coady and Manley, 2015). Thus, while FUS affects transcription of its target genes, it may also alter the stability of target mRNAs by sequestering them in insoluble cytoplasmic aggregates.

The role of FUS in splicing has been extensively studied for its contribution to the onset and progression of ALS. Mutations in FUS have been implicated in changes in autoregulation (Zhou et al., 2013) and alternative splicing (Nakaya et al., 2013; Qiu et al., 2014; Coady and Manley, 2015) specifically by interacting with U11 snRNA, a noncoding RNA component of the minor spliceosome (Reber et al., 2016). In the nucleus, FUS induces the splicing of its own exon 7, reducing RNA stability and leading to nonsense-mediated decay (NMD; Lagier-Tourenne et al., 2012; Zhou et al., 2013). Due to mutations in the NLS, FUS mutants are unable to re-enter the nucleus and the splicing of exon 7 does not occur, ultimately disrupting the negative feedback loop and increasing FUS transcript and protein levels (Zhou et al., 2013).

As evidenced by mouse and human FUS CLIP-seq analysis, FUS binds preferentially to the beginning of long introns, and this binding progressively decreases toward the 3′ end of pre-mRNAs in a “sawtooth”-like pattern (Lagier-Tourenne et al., 2012). Expression of mutant FUS in mammalian cells induces changes in alternative splicing. Analysis by RT-PCR showed that expression of mutant FUS variants leads to a decrease in the e2 isoform of *MECP2* mRNA and a 15-fold increase in the expression of the e1 isoform. This is particularly interesting considering that knock-down of MECP2 has previously been shown to cause altered expression of genes with known synaptic functions (Gabel et al., 2015). Furthermore, depletion of FUS in mammalian cells results in differential splicing of over 400 introns, likely due to its interaction with the minor spliceosome component, U11 snRNA. Moreover, mutations in FUS cause U11 snRNA to localize within cytoplasmic granules (Reber et al., 2016) suggesting a role for FUS in alternative splicing by regulating minor introns. Interestingly, FUS may also play a role in the formation of Gems, which are crucial for spliceosome maturation of Yamazaki et al. (2012). Altogether, FUS is profoundly involved in the splicing of its targets, emphasizing its potential to cause far-reaching dysregulation via its own loss of function.

#### mRNA Transport

Proteomic analyses of purified GST-FUS complexes identified insulin-like growth factor II mRNA-binding protein 1 (IMP1) as a protein partner of FUS (Kamelgarn et al., 2016). IMP1 is involved in recruitment of target mRNAs to cytoplasmic complexes and accompanies them during transport to their final destination in neurons (Kamelgarn et al., 2016). FUS mutants sequester IMP1 in cytoplasmic inclusions (Kamelgarn et al., 2016) suggesting the possibility that in the context of FUS mutations, the localization of IMP1’s own mRNA targets are altered. Potential targets include growth-associated protein 43 (GAP-43), which is important for neuronal regeneration (Donnelly et al., 2011) and may in part explain FUS dependent phenotypes in ALS. Moreover, in mammalian cells, cytoplasmic FUS inclusions affect the localization of the *Ddr2* and *Kank2* mRNAs, whose transport is dependent on the adenomatous polyposis coli (APC) protein. Mechanistically, both the loss of detyrosinated microtubules and the sequestration of *kinesin-1* mRNA and protein into FUS granules contribute to the altered transport and localization of specific RNAs (Yasuda et al., 2017).

#### Stress Granules

As with other RNA-binding proteins involved in ALS, FUS is incorporated into stress granules. Unlike TDP-43, mutant but not wild-type FUS protein, localizes to stress granules in response to oxidative stress or heat shock in mammalian cells, and in zebrafish embryo spinal neurons (Bosco et al., 2010). Studies in HeLa cells indicated that the RGG rich Zn finger motif is the most important RNA-binding domain of FUS required for recruitment to stress granules (Bentmann et al., 2012). The glycine-rich and the RRM domains have a minor contribution, whereas the prion-like glutamine-rich domain is dispensable for recruitment to stress granules (SGs). Studies in neuronal cell lines have corroborated this by showing that RNA-binding-incompetent FUS 4F-L does not incorporate into cytoplasmic stress granules (Daigle et al., 2013). Moreover, FUS R518K, which normally localizes to cytoplasmic stress granules, loses this ability after loss of its RNA-binding capacity (FUS 4F-L R518K; Daigle et al., 2013).

Mutations in FUS lead to an increase in the size and number of stress granules in mammalian cells relative to cells expressing wild-type FUS (Baron et al., 2013). Furthermore, studies in human cells and mouse embryonic stem cells support the formation of FUS-positive membraneless organelles, otherwise known as liquid droplets *in vivo* (Murakami et al., 2015; Patel et al., 2015). The “evolution” of these droplets, as tested by fusion rate, is sped up by mutations in FUS (Patel et al., 2015). Although the precise mechanism by which FUS is incorporated into SGs remains unknown, sequestration of FUS and its protein partners into these cytoplasmic organelles appears to contribute to disease pathogenesis. Supporting this view are recent findings that overexpression of Pur-alpha blocks the cytoplasmic mislocalization of mutant FUS and mitigates neuronal toxicity (Daigle et al., 2016).

## C9orf72 RNA Based Mechanisms in ALS

In 2011, GGGGCC (G_4_C_2_) repeat expansions in the *C9orf72* (*C9*) gene were found to be causative of ALS and FTD (DeJesus-Hernandez et al., 2011; Renton et al., 2011). *C9* repeat expansions are the most common cause of FALS/FTD and are also found in sporadic cases (Ling et al., 2013). Currently, it is thought that *C9* toxicity results from multiple mechanisms including haploinsuffiency, RNA gain of function, and DPRs generated by RAN translation (Taylor et al., 2016; Freibaum and Taylor, 2017; Gendron and Petrucelli, 2017; Wen et al., 2017). Indeed, G_4_C_2_ repeat expansions have been shown to form RNA foci as well as five DPR species (poly-GA, poly-GR, poly-GP, poly-PA and poly-PR), which are thought to contribute to toxicity at least in part by sequestering other proteins and RNAs (Ling et al., 2013; Wen et al., 2017). Since its discovery, considerable effort has been made towards the elucidation of mechanisms underlying *C9* mediated toxicity with multiple studies pointing towards a dysregulation of RNA metabolism as a significant contributor to *C9* disease pathogenesis.

### Gene Expression and Splicing

RNA seq experiments have revealed that *C9* repeat expansions result in altered gene expression in different brain regions from ALS patients as well as lymphoblastoid cell lines (Lagier-Tourenne et al., 2013; Sareen et al., 2013; Cooper-Knock et al., 2015; Prudencio et al., 2015). Interestingly, while some of these changes are distinct, several alterations in RNA processing and metabolism were shared among *C9* and sALS cases (Prudencio et al., 2015) suggesting the potential for mechanistic overlap among all ALS cases. In addition, knock-down of *C9* results in decreased TIA-1 and HuR transcript levels (Maharjan et al., 2017) suggesting a role for *C9* in the transcription of stress granule genes. Furthermore, DPR inclusions have been shown to colocalize with both heterochromatin and the transcriptional repressor H3K9me2 (Schludi et al., 2015), suggesting that *C9* generated dipeptides may play a more direct role in regulation of gene expression.

Of the genes differentially expressed in lymphoblastoid cells derived from *C9* ALS patients, a significant fraction are involved in RNA splicing (Cooper-Knock et al., 2015). Furthermore, although it has been suggested that the overall number of splicing events remains unchanged, the normal profile of alternative splicing events appears to be altered in *C9* repeat expansion models (Cooper-Knock et al., 2015; Prudencio et al., 2015). One possible mechanistic explanation for altered splicing in *C9* ALS stems from the observation that *C9* RNA foci sequester the RNA binding protein and known regulator of splicing, hnRNP H (Lee et al., 2013; Conlon et al., 2016). As a result, RNA targets of hnRNP H are misspliced in ALS patient brain samples (Conlon et al., 2016), which may contribute to disease progression.

Among the many gene expression changes in *C9* ALS, there are alterations in genes linked to both ALS and RNA metabolism such as FUS (Prudencio et al., 2015). Furthermore, in induced pluripotent stem cell (iPSC) models of *C9*-ALS, there is a redistribution of TDP-43 from the nucleus to the cytoplasm (Zhang et al., 2015), which may in turn alter TDP-43’s nuclear function. In addition, mass spectrometry experiments show that *C9* RNA foci sequester proteins containing RNA recognition motifs involved in splicing, translation, and nuclear RNA export (Cooper-Knock et al., 2014; Rossi et al., 2015). As a result, *C9*’s effect on gene expression could be two fold; either by directly regulating gene expression including transcription and splicing or via RNA foci mediated sequestration of proteins involved in RNA metabolism.

### Stress Granules and Translation

More recently, *C9*, like TDP-43 and FUS, has been linked to RNA stress granules (Lee et al., 2016; Maharjan et al., 2017). Multiple RNA binding proteins, mitochondrial ribosomal proteins, and components of stress granules, notably many of which contain Low-complexity domains (LCDs), were identified in poly-GR and poly-PR dipeptide repeat complexes in mammalian cells (Lee et al., 2016; Lin et al., 2016; Lopez-Gonzalez et al., 2016). In *Drosophila*, many of these stress granule proteins exhibit functional interactions with dipeptide repeats by enhancing or suppressing neurodegeneration in the fly eye neuroepithelium (Lee et al., 2016). Upon induction of cellular stress in mammalian cells or primary cortical neurons, C9 colocalizes with G3BP1, HuR and TIA-1 in RNA stress granules (Maharjan et al., 2017). Furthermore, overexpression of *C9*, either pathogenic G_4_C_2_ repeat expansions, or poly-GR/poly-PR dipeptides induces the spontaneous formation of stress granules (Rossi et al., 2015; Lee et al., 2016). Notably, both phosphorylation of eIF2α and G3BP are necessary for spontaneous SG assembly in cells expressing poly-PR. Mechanistically, poly-PR undergoes liquid-liquid phase transition and subsequently causes the phase separation of proteins involved in RNA metabolism. Ultimately, compared to stress granules formed by arsenite treatment, poly-PR induced stress granules contain more TDP-43 and Ataxin-2 (Boeynaems et al., 2017). Although overexpression of *C9* does not alter stress granule assembly or disassembly (Maharjan et al., 2017), expression of poly-GR or poly-PR dipeptides in mammalian cells results in a persistence of stress granules (Lee et al., 2016) suggesting that stress granule disassembly may be compromised. Interestingly, *C9* protein itself is required for stress granule formation as CRISPR/Cas9 knockdown of *C9* expression results in reduced stress granule formation following stress induction in mammalian cells (Maharjan et al., 2017). Mechanistically, knock-down of *C9* reduces protein and transcript levels of the stress granule proteins TIA-1, HuR and G3BP1 (Maharjan et al., 2017) providing an explanation for its effect on stress granule formation.

A key component of stress granules is non-translated mRNA (Anderson and Kedersha, 2009) therefore alterations in stress granule formation or dynamics likely affects translation. Consistent with this scenario are findings that poly-GR and poly-PR dipeptides form a complex with ribosomal proteins and translation initiation and elongation factors such as TDP-43, eIF3A, eEF1A, RPL7A (Kanekura et al., 2016; Lee et al., 2016). The interaction between poly-PR and RNA binding proteins is partially dependent on RNA (Lee et al., 2016), indicating that these interactions likely occur within RNA—Protein complexes. In addition, *C9* RNA foci associate with translation regulators (Rossi et al., 2015) while expression of poly-GR and poly-PR dipeptide repeats appears to inhibit global translation (Kanekura et al., 2016; Lee et al., 2016). Interestingly, poly-GR or poly-PR may also impact ribosome biogenesis as cells expressing either GFP-GR or GFP-PR have a reduction in total rRNA potentially due to the impact of these DPRs on the biophysical properties and dynamics of the nucleolus through their interaction with NPM1 and NCL, two proteins that function in ribosome biogenesis (Lee et al., 2016). Together, these studies suggest that the association of toxic products of *C9* with stress granules influences mRNA translation although the precise mechanism remains unknown.

### Nucleocytoplasmic Transport and RNA Localization

Multiple groups have identified defects in nucleocytoplasmic transport as a pathogenic mechanism in *C9* ALS models (Freibaum et al., 2015; Jovičić et al., 2015; Zhang et al., 2015; Boeynaems et al., 2016). Not only do many genes involved in nucleocytoplasmic transport alter *C9* toxicity, but the nuclear–cytoplasmic ratio of key regulators of this transport process are altered in *C9* models of ALS (Zhang et al., 2015). Interestingly, nuclear pore proteins are also mislocalized in sALS suggesting that disruptions in nucleo/cytoplasmic transport and the integrity of the nuclear pore may underlie the pathogenesis of all ALS cases (Shang et al., 2017). Additionally, *C9* repeat expansions cause nuclear mRNA accumulation (Freibaum et al., 2015; Rossi et al., 2015). Poly-PR DPR was shown to bind to the central channel of nuclear pores and result in PolyA mRNA retention within the nucleus (Shi et al., 2017). Thus, it is possible that *C9* DPR proteins may further impede mRNA transport throughout other areas of the cell via disruptions in nucleo/cytoplasmic transport. It is hypothesized that these defects may contribute to the mislocalization of key proteins such as TDP-43 early in the cascade of pathogenic events (Zhang K. et al., 2016). In turn, this could further impact RNA metabolism. Supporting this notion, cytoplasmic poly-GA expression impairs importin-α mediated nuclear transport ultimately resulting in altered nuclear import of a reporter construct containing the TDP-43 NLS (Khosravi et al., 2017). In addition, poly-GA aggregates sequester nucleocytoplasmic transport proteins (Zhang Y. J. et al., 2016), which may in turn compound effects on this process which is critical for cellular survival.

G_4_C_2_ repeat RNA itself localizes to neuritic RNP granules that contain FMRP resulting in branching defects. Furthermore, expression of the transport granule components and translational regulators FMRP and CPEB3, as well as PSD-95, a known FMRP target are increased in *C9* iPSC neurons which suggests that G_4_C_2_ incorporation into these granules may affect neuritic mRNA localization and local translation (Burguete et al., 2015).

## Concluding Remarks

RNA processing defects have emerged as key and early events in the pathogenesis of ALS. TDP-43, FUS and C9orf72 mutations and/or pathology share common defects in gene expression and post-transcriptional gene regulation. Most importantly, alterations at any level of RNA processing ultimately affect protein expression or localization and contribute to disease progression. Furthermore, although TDP-43, FUS and *C9orf72* may affect all aspect of RNA metabolism, it is important to note that the effects are likely multi-factorial. Specifically, these disease associated loci may alter the expression of genes involved in RNA processing thus, compounding their effects on RNA metabolism.

Although RNA processing defects seem to play a central role in ALS pathogenesis, the precise mechanisms by which they occur remain largely unknown particularly in regards to post-transcriptional RNA processing. Although the ribostasis hypothesis is supported by several reports from multiple laboratories, it has yet to be demonstrated that individual RNA SGs “evolve” into pathological aggregates. As suggested by recent reports, it is plausible that SGs form in a manner similar to liquid droplets. However, as these studies focus on proteins with LCDs, the impact of RNA on this process remains to be determined. A more detailed understanding of the different types of RNA granules, aggregates and their relationship is needed. To further understand mechanisms underlying ALS it will also be important to understand how each of these ALS associated genes and their mutant variants regulate, and contribute to dysregulation of specific aspects of RNA metabolism. Identifying additional RNA targets whose expression is altered in disease and the cellular processes affected by this dysregulation is also needed to understand disease pathomechanisms and perhaps the reason(s) for the indistinguishable clinical presentations across fALS and sALS. In addition, it would be interesting to determine common and divergent RNA targets in different models of ALS. This will assist in the development of therapeutic strategies that may be effective for all or defined subsets of ALS patients.

## Author Contributions

ANC, BLZ and DCZ wrote the manuscript. ANC focused on TDP-43 and C9, and made the figure. BLZ focused on the FUS section.

## Conflict of Interest Statement

The authors declare that the research was conducted in the absence of any commercial or financial relationships that could be construed as a potential conflict of interest.
